# A treatment-site-specific evaluation of commercial synthetic computed tomography solutions for proton therapy

**DOI:** 10.1016/j.phro.2024.100639

**Published:** 2024-09-01

**Authors:** Ping Lin Yeap, Yun Ming Wong, Kang Hao Lee, Calvin Wei Yang Koh, Kah Seng Lew, Clifford Ghee Ann Chua, Andrew Wibawa, Zubin Master, James Cheow Lei Lee, Sung Yong Park, Hong Qi Tan

**Affiliations:** a Division of Radiation Oncology, National Cancer Centre Singapore, Singapore; b Department of Oncology, University of Cambridge, United Kingdom; c Division of Physics and Applied Physics, Nanyang Technological University, Singapore; d Oncology Academic Clinical Programme, Duke-NUS Medical School, Singapore

**Keywords:** Proton therapy, Adaptive radiotherapy, Synthetic CT, Cone-beam CT

## Abstract

**Background and purpose:**

Despite the superior dose conformity of proton therapy, the dose distribution is sensitive to daily anatomical changes, which can affect treatment accuracy. This study evaluated the dose recalculation accuracy of two synthetic computed tomography (sCT) generation algorithms in a commercial treatment planning system.

**Materials and methods:**

The evaluation was conducted for head-and-neck, thorax-and-abdomen, and pelvis sites treated with proton therapy. Thirty patients with two cone-beam computed tomography (CBCT) scans each were selected. The sCT images were generated from CBCT scans using two algorithms, Corrected CBCT (corrCBCT) and Virtual CT (vCT). Dose recalculations were performed based on these images for comparison with “ground truth” deformed CTs.

**Results:**

The choice of algorithm influenced dose recalculation accuracy, particularly in high dose regions. For head-and-neck cases, the corrCBCT method showed closer agreement with the “ground truth”, while for thorax-and-abdomen and pelvis cases, the vCT algorithm yielded better results (mean percentage dose discrepancy of 0.6 %, 1.3 % and 0.5 % for the three sites, respectively, in the high dose region). Head-and-neck and pelvis cases exhibited excellent agreement in high dose regions (2 %/2 mm gamma passing rate >98 %), while thorax-and-abdomen cases exhibited the largest differences, suggesting caution in sCT algorithm usage for this site. Significant systematic differences were observed in the clinical target volume and organ-at-risk doses in head-and-neck and pelvis cases, highlighting the importance of using the correct algorithm.

**Conclusions:**

This study provided treatment site-specific recommendations for sCT algorithm selection in proton therapy. The findings offered insights for proton beam centers implementing adaptive radiotherapy workflows.

## Highlights

• Proton therapy dose accuracy is affected by daily anatomical changes.
• Two synthetic computed tomography algorithms were evaluated.
• 30 patients from head-and-neck, thorax-and-abdomen, and pelvis sites.
• Site-specific synthetic algorithm selection is crucial for optimal accuracy.

## 1 Introduction

It is well known that proton beams exhibit distinct physical characteristics compared to photons [1], [2]. Protons deposit most of their energy at the end of the path, resulting in the Bragg peak. Intensity-modulated proton therapy is therefore capable of attaining comparable tumour control probabilities to conventional X-ray radiotherapy, while achieving reduced radiation doses to surrounding tissues [3], [4]. This facilitates precise and highly conformal delivery of radiation doses to the target, thereby minimising the impact on adjacent healthy tissues; clinical data on the reduced toxicities have been reported in head-and-neck cancer [5], [6], [7], [8], [9], [10].

However, the advantages of a conformal dose distribution come at a cost of an increased sensitivity to daily anatomical changes. Radiotherapy treatment usually requires days of planning using one computed tomography (CT) scan of the patient's anatomy taken during simulation. This treatment is then administered in fractions over several weeks. Throughout the treatment course, patients may experience both inter-fractional and intra-fractional changes in their anatomy [11], [12]. Inter-fractional changes occur between treatment sessions and are typically attributed to factors like weight fluctuations or shifts in organ position, happening over days or weeks. Intra-fractional changes happen within a single treatment session, often due to bodily functions such as breathing or metabolic activity, occurring over a matter of seconds to minutes [12], [13]. Consequently, employing the same treatment plan based on the initial imaging assessment for all sessions can result in discrepancies between the planned dose and the actual dose received by the target and organs-at-risk (OARs) [14], [15], [16].

Adaptive radiotherapy aims to tailor the treatment plan according to the patient's anatomy on the day of treatment, thereby enabling accurate dose delivery to the target and enhancing treatment outcomes and quality of life [17], [18]. Cone-beam CT (CBCT) scans, typically taken during treatment for patient positioning, suffer from poorer image quality and inaccurate CT numbers [19], hence disabling direct dose evaluation. To achieve accurate adaptation, it is necessary to generate a planning quality CT scan from a CBCT scan.

Various methods have been developed to enable CBCT-based synthetic CT (sCT) image generation in commercial treatment planning systems [20], [21]. This ranges from simple CT number correction curves [22] to advanced methods that employ deformable image registration (DIR) to deform a planning CT (pCT) to the treatment CBCT [23], [24], [25]. More recently, there has been increasing research on deep learning based neural networks [26] such as generative adversarial networks (GANs) and U-nets [27], [28], [29], [30]. Various studies have also been conducted to evaluate different subsets of these methods [31], [32].

We investigated two sCT generation algorithms in this work: the corrected CBCT method and the virtual CT method. Thing et al. evaluated both algorithms on 60 patients treated with photon beams, and reported excellent dose volume histogram (DVH) agreement between the sCT and reference CT images [33]. Chang et al. evaluated both algorithms on 23 patients treated with proton therapy, and proposed a framework to identify the optimal sCT algorithm to use based on efficiency and DVH accuracy [34]. In this work, we evaluated the two algorithms based on treatment-site-specific dose recalculation performances in proton therapy. We provided recommendations for the optimal sCT algorithm of the two to use for head-and-neck, thorax-and-abdomen, and pelvis sites for proton therapy.

## 2 Material and methods

### 2.1 RayStation synthetic CT algorithms

In RayStation 11B (RaySearch Laboratories, Stockholm, Sweden), the introduction of two algorithms made it possible to generate sCT images in under 10 s with accurate CT numbers, enabling accurate dose computation.

In the Corrected CBCT (corrCBCT) algorithm, a conversion from the CBCT intensity scale to the planning-quality reference CT (refCT) Hounsfield Unit (HU) intensity scale is first created, then a correction map that removes low frequency artefacts for each voxel in the CBCT is utilised. These two stages are performed iteratively until convergence. This algorithm can be applied to all CBCTs, without any calibration required. However, if the original CBCT is of poor quality, some artefacts may remain. As the two stages do not affect the patient’s anatomy, there is no risk of changes to the CBCT geometry. In the event of limited field-of-view (FOV), voxels from outside the FOV are copied from the deformed planning CT to the CBCT.

In the Virtual CT (vCT) algorithm, a refCT is first deformed to the CBCT geometry, and the mismatching low-density tissues (e.g. air or lung) in the refCT or the CBCT are then substituted with values from the corrCBCT. As the vCT is mostly a deformed CT, it will generally be of CT quality and hence the additional dose recalculation errors due to image quality will in principle be small. However, the accuracy of the vCT is highly dependent on the DIR process, which can result in imperfect anatomical representation and sometimes physically unrealistic deformations [35].

### 2.2 Patient selection and imaging

This study was approved by SingHealth Institutional Review Board. 30 patients (10 per treatment site) with two CBCT scans per patient acquired on different days, treated with curative intent, were selected for this study. The two days were chosen to be the first fraction and during mid-treatment. The three treatment sites were selected based on the unique CBCT imaging protocols – 1) head-and-neck, 2) thorax-and-abdomen and 3) pelvis. For head-and-neck and thorax-and-abdomen sites, repeat CT (reCT) scans were taken weekly for dose evaluation, while for pelvis site, reCT scans were taken on demand. CBCT scans were taken using the Hitachi ProBeat (Hitachi, Tokyo, Japan) on-board imaging system before each fraction for patient positioning.

The Hitachi ProBeat proton therapy system at the National Cancer Centre Singapore uses a synchrotron spot scanning delivery technique with 98 discrete energy layers ranging from 70.2 to 228.7 MeV. All the gantries were equipped with CBCT which had a source-to-imager distance of 1.6 m. The CBCT had small and large FOV modes which corresponded to full-fan (25 cm FOV) and half-fan acquisitions (48 cm FOV), respectively. Both modes had the same scan lengths of 25 cm. CT simulation scans were acquired using Siemens SOMATOM X.cite (Siemens Healthineers, Forchheim, Germany) or GE Revolution (GE Healthcare, Milwaukee, WI, USA) CT systems. All CT and CBCT scans were acquired at 120 kVp, except head-and-neck CBCT scans which were acquired at 100 kVp. Only large FOV CBCTs are included for pelvis and thorax-and-abdomen while only small FOV CBCTs are used for head-and-neck. The FOV is chosen to ensure the CBCT image is not truncated. The exposure settings for individual treatment sites were optimized for best contrast-to-noise ratio (CNR) in an anthropomorphic phantom during the commissioning phase.

Monte Carlo dose calculations with robust optimization were performed in RayStation 2023A with a grid spacing of 3.0 mm or smaller. Robust optimization was applied with a range uncertainty of 3.5 % and a setup uncertainty of 3.0 mm for head-and-neck and prostate plans, and 5.0 mm for thorax-and-abdomen plans. The thorax-and-abdomen treatment plan angles varied according to the target’s location. The prostate plans consisted of lateral fields for the primary prostate target and two posterior oblique fields for the pelvic lymph nodes (if treated). The head-and-neck patients were mainly nasopharyngeal carcinoma. The primary tumor and lymph nodes were treated with four fields comprising anterior and posterior oblique fields. An additional anterior field was used to treat nodes in the lower neck.

### 2.3 Evaluation

**Fig. 1:**
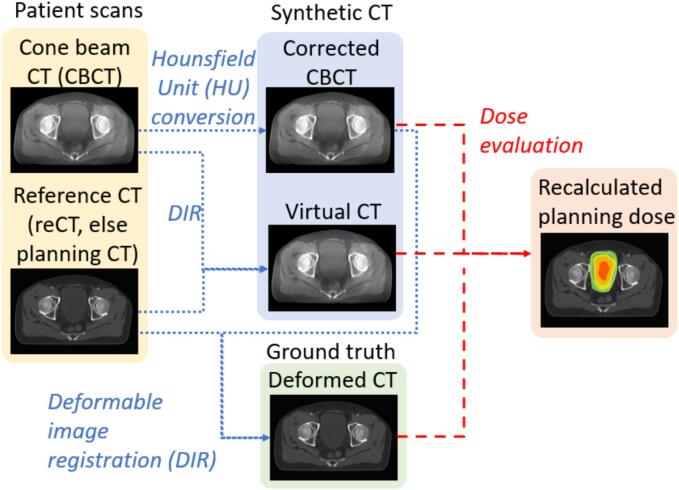
Schematic of the evaluation and validation process of synthetic computed tomography (CT) in RayStation.

For each of the two CBCT scans, the reCT acquired on the same day as the CBCT was used as the refCT. This approach was applied for the CBCTs from head-and-neck and thorax-and-abdomen treatment sites as there was a weekly reCT for these cases. If no reCT scans were acquired (which was the case for most pelvis patients), the pCT acquired during simulation was used as the refCT instead. Only four out of twenty prostate CBCTs used reCT as refCT. Next, a corrCBCT was generated through RayStation using the Corrected CBCT algorithm, and a vCT was generated using the Virtual CT algorithm by deformably registering the refCT to the CBCT. In addition, to account for anatomical changes, the refCT was also deformably registered to the corrCBCT to generate a deformed reference CT (dCT), which was used as the “ground truth” CT here. Finally, the radiotherapy dose was recalculated on the corrCBCT and the vCT using the same radiotherapy plan and compared to the dose recalculated on the dCT. The full schematic of this workflow is shown in Fig. 1. The target and OAR structures were mapped from the original pCT or reCT to the sCTs using the same DIRs. All the structures in the sCTs were visually validated to ensure the DVHs were representative of the structure.

Three different metrics were used to evaluate the quality of the two sCTs. These metrics included the 2 %/2 mm and 1 %/1 mm gamma passing rates (GPRs) [36] and the mean percentage dose differences relative to the prescription dose which was the prescribed dose to the primary target. For the gamma analysis, the low dose thresholds were set at 10 %, 50 %, and 80 %, with a focus on 80 % to investigate dose differences in the high dose regions in the target. We also evaluated the DVH differences in the clinical target volumes (CTV) and OARs. The target DVHs were CTV D95 and D98. The OAR DVHs were assessed differently for each of the three sites. The mean right and left parotid doses, and the maximum spinal cord, optic chiasm and brainstem were selected for head-and-neck. The mean heart and oesophagus doses and the maximum spinal cord and heart doses were selected for the thorax-and-abdomen sites. The maximum doses of bladder and rectum were selected for pelvis site. Wilcoxon signed-rank tests were performed for all statistical comparison. A two-tailed P<0.05 was regarded as significant in this study.

## 3 Results

### 3.1 Synthetic CT algorithms

**Fig. 2:**
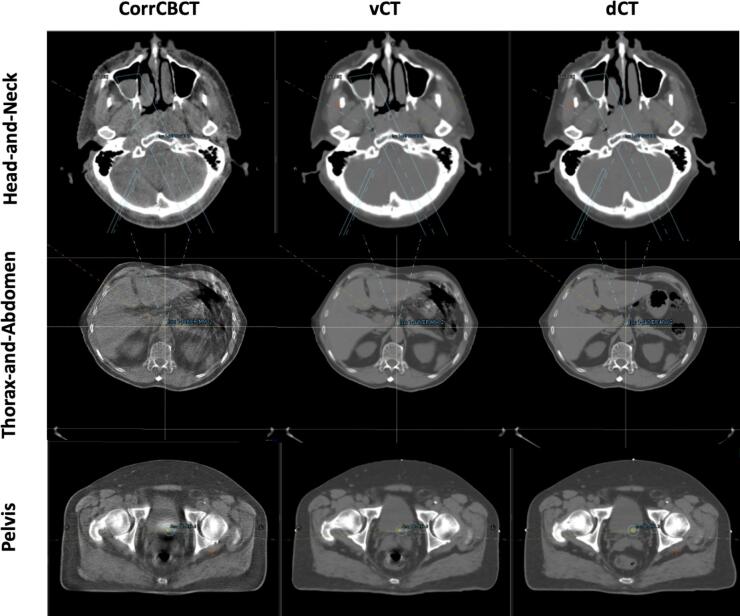
Comparison of corrected CBCT (corrCBCT), virtual CT (vCT), and deformed reference CT (dCT) images for the three treatment sites.

As seen in Fig. 2, corrCBCT, unlike vCT, did not remove the CBCT artefacts such as the streaking from the bowel and rectal gases or the shading artefacts. The corrCBCT images also looked “grainier” compared to the vCT. Clear anatomical differences between the dCT and sCT were observed in the rectum and bowel area for the pelvis and thorax-and-abdomen treatment sites, respectively. Due to the intense streaking artefacts in the CBCT in these regions, it was challenging to achieve “CT-quality” in the sCT in those regions.

### 3.2 Evaluation

The mean percentage dose difference for corrCBCT yielded a closer dose agreement to the dCT for head-and-neck cases, with a mean percentage dose discrepancy of 0.6 % in the high dose region (i.e., >80 % of maximum dose; Table 1). For the pelvis cases, the vCT algorithm yielded a closer dose agreement, with a mean percentage dose discrepancy of 0.5 % in the high dose region. For the thorax-and-abdomen cases, the vCT algorithm yielded a closer dose agreement, with a mean percentage dose discrepancy of 1.3 % in the high dose region.

**Table 1:** Mean percentage dose differences and gamma passing rates (GPR) between the sCT and dCT scans for the 3 sites. Values in parenthesis refer to 1 standard deviation.

	corrCBCT	vCT
Head-and-Neck		
Dose differences (80 % th)/%	0.6 (0.08)	0.6 (0.14)
Dose differences (50 % th))/%	0.9 (0.21)	0.9 (0.24)
2 %/2 mm GPR (80 % th)/%	99.8 (0.23)	99.5 (0.43)
2 %/2 mm GPR (50 % th)/%	99.1 (0.43)	98.9 (0.57)
1 %/1 mm GPR (80 % th)/%	85.2 (2.54)	81.9 (4.12)
1 %/1 mm GPR (50 % th)/%	88.1 (2.54)	85.7 (3.48)

Pelvis		
Dose differences (80 % th))/%	0.8 (0.18)	0.5 (0.08)
Dose differences (50 % th))/%	1.3 (0.55)	0.7 (0.23)
2 %/2 mm GPR (80 % th)/%	98.6 (1.52)	99.9 (0.15)
2 %/2 mm GPR (50 % th)/%	98.8 (1.05)	99.7 (0.34)
1 %/1 mm GPR (80 % th)/%	71.4 (9.50)	83.3 (6.59)
1 %/1 mm GPR (50 % th)/%	89.4 (9.50)	93.7 (1.87)

Thorax-and-Abdomen		
Dose differences (80 % th))/%	1.4 (0.95)	1.3 (1.19)
Dose differences (50 % th))/%	1.7 (0.91)	1.4 (0.74)
2 %/2 mm GPR (80 % th)/%	93.9 (7.86)	95.3 (4.57)
2 %/2 mm GPR (50 % th)/%	94.9 (3.35)	94.5 (4.95)
1 %/1 mm GPR (80 % th)/%	69.1 (15.23)	73.0 (10.44)
1 %/1 mm GPR (50 % th)/%	78.0 (15.23)	77.9 (12.92)

**Fig. 3:**
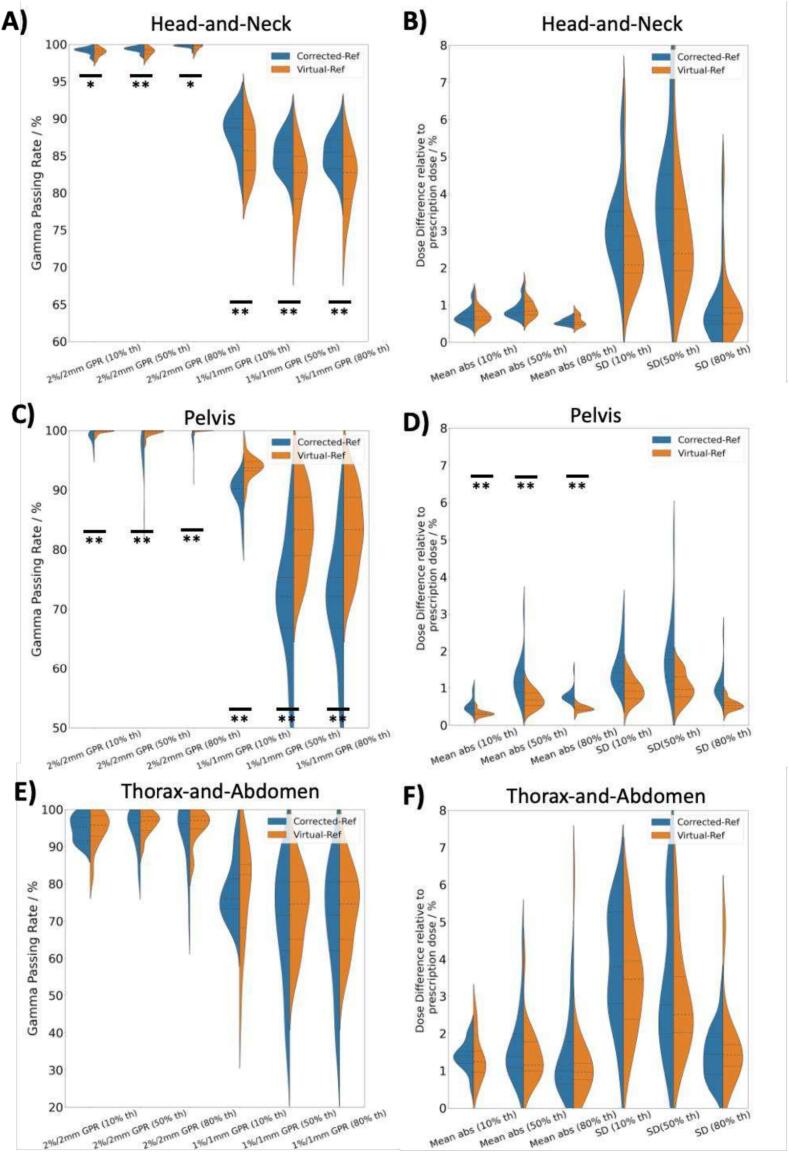
Comparison of the GPRs and dose differences between corrCBCT and vCT with the dCT. Sub-figures A, C and E show the GPRs between the sCT and dCT for 1 %/1 mm and 2 %/2 mm criteria under three different dose thresholds of 10, 50 and 80 %. Sub-figures B, D and F show the mean percentage difference between the sCT and dCT together with the standard deviation (SD) of the voxel-wise percentage dose difference. 1 asterisk signifies p < 0.05, while 2 asterisks signify p < 0.01 for the Wilcoxon signed-rank tests.

A comparison of the GPRs in Fig. 3**A, 3C and 3E** showed that the GPRs were significantly higher for corrCBCT in head-and-neck and vCT in pelvis compared to the alternative method, whereas the results were indeterminate for thorax-and-abdomen treatment site. This conclusion was similar for both 1 %/1 mm and 2 %/2 mm criteria.

The largest dose differences were encountered in the thorax-and-abdomen cases, with the mean percentage dose differences as high as 1.7 % in the corrCBCT images. The 2 %/2 mm GPR for thorax-and-abdomen cases all fall below 96 %.

**Fig. 4:**
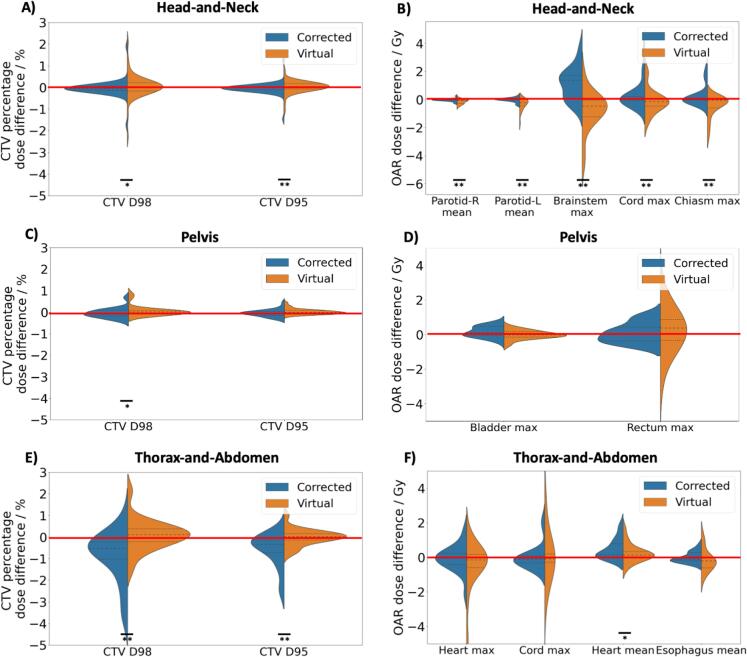
Comparison of the CTV and OAR DVH differences. Figure A, C and E show the percentage dose difference of D98 and D95 for the CTV. Figure B, D and F show the absolute dose difference between various DVH metrics for the OARs. 1 asterisk signifies p < 0.05, while 2 asterisks signify p < 0.01. The red line represents zero difference. (For interpretation of the references to colour in this figure legend, the reader is referred to the web version of this article.)

As seen in Fig. 4, statistically significant difference in all the CTVs and OAR dose metrics were observed for head-and-neck treatment site. On the other hand, only CTV D98 showed statistically significant difference between vCT and corrCBCT in the pelvis site, despite the GPRs and percentage dose discrepancy indicating otherwise. The thorax-and-abdomen site showed significant difference for CTV D98, CTV D95 and heart mean dose.

## 4 Discussion

In this work, we have detailed an experimental design to evaluate the dose recalculation performances of synthetic CT scans generated with a commercial treatment planning system. The sCT scans for head-and-neck and pelvis sites showed clinically acceptable DVH agreement with the dCT (ground truth in this study). We have also provided recommendations on the optimum sCT algorithm to use for specific sites treated with proton therapy.

The GPR results in Fig. 3 conclusively showed that corrCBCT and vCT work best for head-and-neck and pelvis treatment site, respectively. Nonetheless, the 2 %/2 mm GPR for head-and-neck and pelvis cases achieved > 98 % for both algorithms, which is consistent with other reported performances [16]. Unlike the head-and-neck and thorax-and-abdomen sites, the pelvis site showed significantly lower mean percentage dose difference for vCT compared to corrCBCT. A closer look at the standard deviation of the dose errors showed that the pelvis site had the smallest standard deviation compared to the other two sites. This could be attributed to the heterogenous anatomy in the other two treatment sites which caused a large spread in the dose errors. The dose recalculation errors between the sCT and the dCT could also be attributed to sCT algorithm errors, such as errors in CBCT-to-CT HU conversions and errors from assigning dCT as the ground truth. It was impossible to decouple both contributions but nonetheless, the overall mean percentage dose errors were low enough (less than 1 %) for the sCT to be used clinically to assess daily target coverage.

Fig. 4 showed that it is important that the correct sCT method is used for head-and-neck as there was a statistically significant and systematic difference between the clinical DVHs (for both targets and OARs) between using the vCT or corrCBCT. Allen et al. generated sCTs of head-and-neck cancer patients using various DIR algorithms on the VelocityAI v4.1 software (Varian Medical Systems, CA), and similarly reported that the choice of algorithm can affect dose calculation accuracy [37]. The pelvis results in Fig. 4 showed that using the correct method was necessary to avoid the systematic difference in the CTV D98. The sCT solutions performed the worst in the thorax-and-abdomen dataset, where the mean percentage errors exceeded 1 % and the GPRs were below 96 % for both sCT solutions, hence it was advised to use the sCT algorithms for plan adaptation with caution for this site. The performance of vCT and corrCBCT were comparable with the vCT showing slightly higher GPR and lower percentage dose error under 80 % dose threshold (no statistical significance as shown in Fig. 3). Nonetheless, as a clinical recommendation, vCT could contain unrealistic physical deformations [31] especially in the thoracic and abdominal regions. Therefore, under the premise that the corrCBCT and vCT scans had comparable performance, corrCBCT was still preferred wherever possible. Since the sCT were unreliable for dose calculation, weekly CT was still enforced for proton treatment in the thorax-and-abdomen region to have an accurate dose review in a weekly setting.

Regardless of the CBCT hardware and the exposure, there were certain similarities in the findings between our work and the two previous works by Thing et al. [33] and Chang et al. [34]. Our result agreed with the findings by Chang et al. [34] (did not evaluate for pelvis) that we should use corrCBCT for head-and-neck site and agreed with the findings by Thing et al. [33] (did not evaluate for head-and-neck) that vCT should be used for pelvis. All our results showed that it is possible to achieve a 2 %/2 mm GPR greater than 97 % for the two above-mentioned treatment sites. However, both authors did not agree on the sCT method for thorax regions (Chang et al. [34] recommended vCT and Thing et al. [33] vice versa). Taasti et al. found that the vCT algorithm produced more false negatives than the corrCBCT approach in lung cancer patients, where a false negative happened when the reCT flagged a plan adaptation but the sCT did not [38]. Even though our thorax-and-abdomen result showed a slightly higher dose concordance for vCT compared to corrCBCT, the results were not great in general and sCT should be still used with caution in this site. Due to the disagreement on the sCT method for this treatment site, each centre should perform an in-depth evaluation of sCT for the thorax-and-abdomen site with their own CBCT and delivery system.

One consistent finding in our and other authors’ work was that corrCBCT worked best for a thinner scan volume and vCT for a thicker one. The size of the treatment site or patient size directly affected the CBCT image qualities as a larger radiological length would result in more scattering. Since corrCBCT did not remove any CBCT artefact (scatter, ring, shading, streaking), it was expected that the corrCBCT would be less accurate with increasing imaging artefacts. The exposure settings of the CBCT were also expected to affect the corrCBCT quality as the exposure setting would directly affect the signal-to-noise ratio (SNR) of the CBCT and thus the corrCBCT as well (lower exposure setting would decrease the SNR).

As the “ground truth” dCT image was derived by deforming the refCT to the corrCBCT, there might be registration errors in the DIR process, even though the dCT images had been visually inspected by a clinically qualified medical physicist. As such, one limitation of this study was the absence of quantitative validation of the “ground truth” images, which might affect the accuracy of the dose evaluation [39]. The ideal ground truth CT data was probably one generated from a CT-on-rails systems which were only available in a small number of centers worldwide [40].

In conclusion, this work examined the dose recalculation performances of two sCT generation algorithms and showed that the choice of sCT generation algorithm could lead to differences in clinical judgements.

## Funding support

Hong Qi Tan is supported by the Duke-NUS Oncology Academic Program Goh Foundation Proton Research Programme (08/FY2023/EX(SL)/163-A218(b)), Clinical & Systems Innovation Support – Innovation Seed Grant (08/FY2022/P2/02-A68).

## CRediT authorship contribution statement

**Ping Lin Yeap:** Validation, Writing – original draft, Writing – review & editing. **Yun Ming Wong:** Validation, Writing – review & editing. **Kang Hao Lee:** Validation, Writing – review & editing. **Calvin Wei Yang Koh:** Validation, Writing – review & editing. **Kah Seng Lew:** Validation, Writing – review & editing. **Clifford Ghee Ann Chua:** Validation, Writing – review & editing. **Andrew Wibawa:** Validation, Writing – review & editing. **Zubin Master:** Validation, Writing – review & editing. **James Cheow Lei Lee:** Validation, Writing – review & editing. **Sung Yong Park:** Supervision, Validation, Writing – review & editing. **Hong Qi Tan:** Conceptualization, Data curation, Formal analysis, Funding acquisition, Investigation, Methodology, Project administration, Visualization, Validation, Writing – review & editing.

## Declaration of competing interest

The authors declare that they have no known competing financial interests or personal relationships that could have appeared to influence the work reported in this paper.
